# Exploration of the anti-diabetic potential of hydro-ethanolic leaf extract of *Koenigia polystachya* L.: an edible wild plant from Northeastern India

**DOI:** 10.1186/s42826-023-00174-3

**Published:** 2023-09-18

**Authors:** Alokali Kiba, Dipankar Saha, Bhrigu Kumar Das

**Affiliations:** Department of Pharmacology, School of Pharmaceutical Sciences, Girijananda Chowdhury University, Azara, Guwahati, Assam 781017 India

**Keywords:** Antidiabetic, Acute oral toxicity, *Koenigia polystachya*, Metformin, Streptozotocin

## Abstract

**Background:**

Globally, medicinal plants are used to treat diseases like diabetes. The present study evaluates the possible antioxidant, acute oral toxicity, the *in-vitro* and *in-vivo* antidiabetic potential of the hydro-ethanolic leaf extract of *Koenigia polystachya* (HELeKP) against beta-cell damage in experimentally induced diabetes mellitus. The DPPH (2,2-diphenyl-1-picrylhydrazine), ABTS [2,2′-azino bis-(3-ethylbenzothiazoline-6-sulfonic acid)], H_2_O_2_ (Hydrogen peroxide), superoxide radical scavenging activity and NO (Nitric oxide) assay estimated the *in-vitro* antioxidant assay of HELeKP. The acute oral toxicity study was evaluated per the OECD (Organization for Economic Cooperation and Development) test guidelines 425. Diabetes was stimulated in rats with a single dose of Streptozotocin (STZ), and after confirmation of diabetes, HELeKP was given orally for 21 days. Blood/serum samples were gathered and examined for biochemical changes, while tissue samples were evaluated for histopathological alterations.

**Results:**

The IC_50_ value of the HELeKP for all the anti-oxidant assays confirms the free radical scavenging activity. The data on acute oral toxicity revealed that the HELeKP used in the study was comparatively very safe. The outcomes of the *in-vivo* study suggested that the extract significantly reduced (*p* < 0.001) the fasting glucose level in STZ-induced diabetic rats. Furthermore, the lipid profile level was significantly normalized (*p* < 0.01, *p* < 0.001) in diabetic rats. The histopathological observation of the pancreas in HELeKP-treated rats showed significant beta-cell restoration.

**Conclusions:**

Based on the outcomes of this study, the HELeKP-treated rats have significant free radical scavenging and anti-diabetic potential. Therefore, it can be recommended as a beneficial functional vegetable for consumption.

## Background

In recent decades, dietary habits and environmental pollution have been increasingly attributed to physiologic disorders and degenerative diseases. Researchers are conducting extensive research on natural products to formulate balanced and functional diets. Although supplements are available, a well-formulated diet is more convenient and practical. Hence, pharmaceutical scientists are also developing plant-derived medicines and formulations to improve efficacy and minimize side effects. It starts with a baseline screening of individual plants from various perspectives to create new drugs or therapeutic medicines. Many plants have been explored for their antioxidant properties to combat diabetes [1]. Considering the broad-spectrum management and low toxicity of natural products for treating diabetes, the World Health Organization (WHO) greatly emphasizes their evaluation.

Diabetic mellitus (DM) affects carbohydrate, fat, and protein metabolisms, and a deficiency in insulin secretion or action marks increases blood glucose levels. As a growing global health concern, diabetes requires proper management, including regular blood glucose monitoring, exercise, a healthy diet, and medication [2]. The International Diabetes Federation (IDF) projects that diabetics will reach 578 million by 2030 and 700 million by 2045 if current trends continue [3].

Diabetes increases free radical damage due to oxidative stress caused by reduced antioxidant activity. In addition to producing superoxide anions, excess blood glucose level produces hydroxyl radicals, damaging proteins, lipids, and deoxyribonucleic acid via the Haber–Weiss reaction. To prevent oxidative stress, antioxidants scavenge free radicals. Hence, the recent focus has been on developing drugs with both antidiabetic and antioxidant activity [1, 4, 5].

*Koenigia Polystachya* Wall. is a rhizomatous, perennial herb and hemicryptophyte originating from the Himalayas, which is commonly dispersed in Asian countries such as India, China, Bhutan, Korea, Nepal, Myanmar, Tibet, Afghanistan, and some areas of Europe. The plant is commonly found in the Northern part of India, such as Himachal Pradesh, Jammu and Kashmir, Uttarakhand, and North Eastern regions of India like Nagaland, Sikkim, Manipur, and Arunachal Pradesh. There are edible shoots and stems with a pleasantly sour taste that can be used as medicinal, vegetable, or ornamental plants [6–8]. The root extract and leaves have been noted to possess antibiotic, antiseptic, antifungal, antinociceptive, antirheumatic, diuretic, and astringent properties, and have been used to treat many diseases such as indigestion, diarrhea, dysentery, and superficial infections such as sores, ulcers, and dermatoses. Additionally, quercetin-3-rhamnoside (quercitrin), total quercetin, and quercetin-3-D-galactoside (hyperoside) are health-promoting phenolic compounds in the plant. Moreover, the plant contains flavanols and catechins, powerful antioxidants that may help prevent oxidative stress-related diseases [8–11]. Hence, this research was designed to evaluate the possible antioxidant, acute oral toxicity, the *in-vitro* and *in-vivo* antidiabetic potential of the hydro-ethanolic leaf extract of *Koenigia polystachya* (HELeKP) against beta-cell damage in experimentally induced diabetes mellitus.

## Results

### Phytochemical screening

The HELeKP was subjected to phytochemical investigation. It was found to have various constituents such as phenolic compounds, carbohydrates, amino acids, flavonoids, tannins, cardiac, anthraquinone and saponin glycosides.

### In-vitro antioxidant activity

The results of the free radical, hydroxyl radical, hydrogen peroxide, superoxide and nitric oxide scavenging activity of the HELeKP, along with their respective standards (Ascorbic acid and Trolox), showed a dose-dependent scavenging activity increase in the leaf extract and standard. The IC_50_ values of the extracts for all the *in-vitro* anti-oxidant profiles expressed in µg/mL are highlighted in Table 1.

**Table 1:** IC_50_ values of the free radical (DPPH), hydroxyl radical (ABTS), hydrogen peroxide (H_2_O_2_), superoxide and nitric oxide (NO) scavenging activity of the hydro-ethanolic extract of the plant, along with their respective standards (Ascorbic acid and Trolox)

DPPH (µg/ml)		ABTS (µg/ml)		H_2_O_2_ (µg/ml)		Superoxide (µg/ml)		NO (µg/ml)	
Extract	Standard^*^	Extract	Standard^#^	Extract	Standard^*^	Extract	Standard^*^	Extract	Standard^*^
303.12	206.98	235.07	150.81	206.46	118.67	265.36	179.55	263.28	197.97

*IC*_*50*_ Inhibitory concentration 50%, *DPPH* 1,1-diphenyl-2-picrylhydrazyl, *ABTS* 2,2′-azino bis-(3-ethylbenzothiazoline-6-sulfonic acid, *H*_*2*_*O*_*2*_ Hydrogen peroxide, *NO* Nitric oxide

*Ascorbic acid

^#^Trolox

### In-vitro antidiabetic assay

The results of the *in-vitro* antidiabetic assay of the HELeKP, along with their respective standards (Acarbose and ascorbic acid), displayed a concentration-dependent antidiabetic activity. The IC_50_ values of the extracts for all the *in-vitro* antidiabetic assays expressed in µg/mL are highlighted in Table 2.

**Table 2:** IC_50_ values of the *in-vitro* anti-diabetic assay (α-amylase, α-glucosidase) of the hydroethanolic extract of the plant, along with respective standard (Acarbose, ascorbic acid)

α-amylase activity (µg/ml)		α-glucosidase activity (µg/ml)	
Extract	Standard (Acarbose)	Extract	Standard (Ascorbic acid)
34.52	66.80	84.39	74.76

### Acute oral toxicity assay

The acute oral limit toxicity dose of the HELeKP at 2000 mg/kg *b.w*. didn’t exhibit any signs of symptoms or mortality throughout the experimental time (14 days), irrespective of gender changes. The body weight didn’t change significantly after using the plant extract (data not shown). During the study, all other parameters, such as fur and skin, eyes, itching, feces consistency, the color of urine, salivation, somatomotor activity, and sleep, were normal throughout the study (Table 3).

**Table 3:** Effect of *Koenigia polystachya* L. extracts on the behavior of rats in acute toxicity studies

Parameters	Observations											
	30 Min		4 h		24 h		48 h		7 days		14 days	
	VC	KPe	VC	KPe	VC	KPe	VC	KPe	VC	KPe	VC	KPe
Fur and skin	N	N	N	N	N	N	N	N	N	N	N	N
Eyes	N	N	N	N	N	N	N	N	N	N	N	N
Salivation	N	N	N	N	N	N	N	N	N	N	N	N
Respiration	N	N	N	N	N	N	N	N	N	N	N	N
Urination (Color)	N	N	N	N	N	N	N	N	N	N	N	N
Feces consistency	N	N	N	N	N	N	N	N	N	N	N	N
Somatomotor activity and behavioral pattern	N	N	N	N	N	N	N	N	N	N	N	N
Sleep	N	N	N	N	N	↑	N	↑	N	↑	N	N
Mucous membrane	N	N	N	N	N	N	N	N	N	N	N	N
Convulsions and tremors	N	N	N	N	N	N	N	N	N	N	N	N
Itching	N	N	N	N	N	N	N	N	N	N	N	N
Coma	A	A	A	A	A	A	A	A	A	A	A	A
Mortality	A	A	A	A	A	A	A	A	A	A	A	A

*VC* Vehicle control, *KPe Koenigia polystachya* L. extract, *N* Normal, *A* Absent, ↑ Increase

### Hematological and biochemical analysis

The data of the hematological analysis exhibited no significant alterations in any parameters compared to the normal rats, as depicted in Table 4. Furthermore, with post limit test dose of the extract, the rats didn’t exhibit any significant alterations in the renal function, liver function, and lipid profile parameters (Table 5).

**Table 4:** Effect of *Koenigia polystachya* L. extract given at a dose of 2000 mg/kg *b.w. p.o.* in different hematological parameters

Parameters	Unit	Vehicle control (0.5% CMC) (Male)	Vehicle control (0.5% CMC) (Female)	*Koenigia polystachya* L. extract (2000 mg/kg) (Male)	*Koenigia polystachya* L. extract (2000 mg/kg) (Female)
Hb	g/dl	11.54 ± 0.097	11.52 ± 0.086	11.42 ± 0.115	11.44 ± 0.092
Total RBC	Millions/mm^3^	4.86 ± 0.024	4.82 ± 0.037	4.80 ± 0.070	4.74 ± 0.050
MCV	fl	55.54 ± 1.120	55.86 ± 0.997	54.74 ± 0.067	54.34 ± 0.365
MCH	pg	23.58 ± 0.080	23.58 ± 0.249	25.30 ± 0.089	25.50 ± 0.219
MCHC	%	36.26 ± 0.040	36.52 ± 0.231	36.50 ± 0.083	35.94 ± 0.211
PCV	%	32.54 ± 0.335	32.64 ± 0.196	32.56 ± 0.181	32.70 ± 0.017
Platelet count	Lac/mm^3^	1.73 ± 0.005	1.78 ± 0.037	1.54 ± 0.007	1.56 ± 0.005
Total leucocyte count	10^3^/µL	9.642 ± 0.016	9.48 ± 0.083	10.05 ± 0.065	10.03 ± 0.046
Polymorphs	%	53.38 ± 0.659	53.34 ± 0.929	53.64 ± 0.263	52.98 ± 0.634
Lymphocytes	%	75.40 ± 1.217	75.56 ± 1.311	75.58 ± 0.845	75.22 ± 1.030
Monocytes	%	2.30 ± 0.035	2.27 ± 0.018	2.30 ± 0.019	2.30 ± 0.016
Eosinophils	%	1.86 ± 0.022	2.14 ± 0.019	1.90 ± 0.016	2.15 ± 0.012

All values are expressed as mean ± SEM (Standard error of the mean), n = 5 animals/group, considering *p* < 0.05 as statistically significant

*CMC* Carboxy methyl cellulose, *Hb* Hemoglobin, *RBC* Red blood cells, *MCV* Mean corpuscular volume, *MCH* Mean corpuscular hemoglobin, *MCHC* Mean corpuscular hemoglobin concentration, *PCV* Packed cell volume, *fl* Femtoliters

**Table 5:** Effect of *Koenigia polystachya* L. extract given at a dose of 2000 mg/kg *b.w. p.o.* in different biochemical parameters

Parameters	Unit	Vehicle control (0.5% CMC) (Male)	Vehicle control (0.5% CMC) (Female)	*Koenigia polystachya* L. extract (2000 mg/kg) (Male)	*Koenigia polystachya* L. extract (2000 mg/kg) (Female)
Urea	mg/dl	0.640 ± 0.024	0.684 ± 0.019	0.678 ± 0.019	0.694 ± 0.019
Creatinine	mg/dl	50.82 ± 0.363	51.00 ± 0.447	51.50 ± 0.389	51.42 ± 0.448
AST	U/L	140.06 ± 1.327	138.73 ± 1.06	142.53 ± 1.391	131.64 ± 1.002
ALT	U/L	37.09 ± 1.67	40.28 ± 1.17	41.30 ± 1.20	44.55 ± 1.032
ALP	U/L	88.07 ± 0.81	94.11 ± 0.94	91.69 ± 1.68	99.50 ± 1.56
TB	mg/dl	0.328 ± 0.102	0.326 ± 0.005	0.326 ± 0.011	0.334 ± 0.010
DB	mg/dl	0.110 ± 0.004	0.110 ± 0.004	0.112 ± 0.003	0.112 ± 0.004
TC	mg/dl	70.40 ± 0.734	69.20 ± 0.374	68.00 ± 0.547	67.40 ± 0.244
TG	mg/dl	80.00 ± 0.316	75.20 ± 2.223	80.20 ± 0.860	75.20 ± 2.354

All values are expressed as mean ± SEM (Standard error of the mean), n = 5 animals/group, considering *p* < 0.05 as statistically significant

*CMC* Carboxy methyl cellulose, *AST* Aspartate transaminase, *ALT* Alanine transaminase, *ALP* Alkaline phosphatase, *TB* Total bilirubin, *DB* Direct bilirubin, *TC* Total cholesterol, *TG* Triglycerides

### Morphological and histopathological analysis

**Fig. 1:**
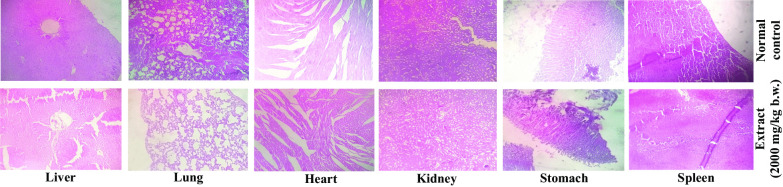
Effect of *Koenigia polystachya* L. extract given at a dose of 2000 mg/kg *b.w. p.o.* on the histology of the vital organs (Liver, Lung, Heart, Kidney, Stomach, and Spleen). The upper panel shows the histology of the normal control animals, and the lower panel depicts the extract-treated group (Magnification: 10 ×)

No significant modifications were observed in the morphology (data not shown) and the histopathological report for all the vital organs, the liver, lungs, heart, kidney, stomach and spleen, in the normal and extract-treated groups (Fig. 1).

### Anti-diabetic study

#### Fasting glucose level

The effect of *Koenigia polystachya* L. extracts given in STZ-induced rats after oral administration on blood glucose level displayed a reduction (*p* < 0.001) in a time-dependent means compared to the STZ-induced rats alone (Table 6).

**Table 6:** Effect of *Koenigia polystachya* L. extract given on blood glucose level (mg/dl) in STZ-induced diabetic rats

Groups	Day 0	Day 7	Day 14	Day 21
NC	82.03 ± 0.1962	81.69 ± 0.1674	81.10 ± 0.2570	81.00 ± 0.2507
STZ-induced diabetic rats	81.49 ± 0.2460	355.4 ± 10.73^***^	390.0 ± 17.54^***^	391.1 ± 17.06^***^
STZ + High dose of the HELeKP	80.91 ± 0.2379	199.7 ± 4.964^###^	165.4 ± 4.640^###^	131.3 ± 6.832^###^
STZ + Low dose of the HELeKP	81.61 ± 0.2709	203.3 ± 6.886^###^	169.6 ± 5.647^###^	151.4 ± 7.080^###^
STZ + Metformin	81.56 ± 0.3240	222.1 ± 3.032^###^	168.1 ± 2.713^###^	123.6 ± 2.497^###^

All values are expressed as mean ± SEM (Standard error of the mean)

*NC* Normal control rats, *STZ* Streptozotocin, *HELeKP* Hydro-ethanolic leaf extract of *Koenigia polystachya*

***p < 0.001 as compared to the normal control rats

^###^*p* < 0.001 as compared to STZ-induced diabetic rats

#### Effect on body weight

The STZ-induced diabetic rats had significantly decreased (*p* < 0.05, *p* < 0.001) their body weight by day 21 compared to the normally treated rats. On the other hand, the treated groups (both the extract and standard) significantly restored (*p* < 0.001) the body weight towards normal compared to the STZ-treated rats (Table 7).

**Table 7:** Effect of *Koenigia polystachya* L. extract given on body weight (g) in STZ-induced diabetic rats

Groups	Day 0	Day 7	Day 14	Day 21
NC	182.5 ± 2.500	186.3 ± 2.631	193.8 ± 2.631	198.8 ± 2.266
STZ-induced diabetic rats	188.8 ± 2.950	172.5 ± 3.134^*^	161.3 ± 2.950^***^	131.3 ± 2.950^***^
STZ + High dose of the HELeKP	188.8 ± 2.950	171.3 ± 3.504	187.5 ± 3.660^###^	196.3 ± 1.830^###^
STZ + Low dose of the HELeKP	190.0 ± 2.673	167.5 ± 3.660	183.8 ± 2.631^###^	191.3 ± 2.950^###^
STZ + Metformin	187.5 ± 3.134	166.3 ± 2.631	187.5 ± 2.500^###^	198.8 ± 2.266^###^

All values are expressed as mean ± SEM (Standard error of the mean)

*NC* Normal control rats, *STZ* Streptozotocin, *HELeKP* Hydro-ethanolic leaf extract of *Koenigia polystachya*

*p < 0.05, ***p < 0.001 as compared to the normal control rats

^###^*p* < 0.001 as compared to STZ-induced diabetic rats

#### Lipid profile

The STZ-treated rats had significantly higher (*p* < 0.001) cholesterol, triglycerides, LDL, VLDL levels and lower HDL levels than the normal rats. Whereas the treated groups significantly restored the lipid levels toward normal (*p* < 0.01, *p* < 0.001) in comparison to the STZ-induced diabetic rats (Table 8).

**Table 8:** Effect of *Koenigia polystachya* L. extract given on lipid profile (mg/dl) in STZ-induced diabetic rats

Groups	TC	TG	HDL	LDL	VLDL
NC	89.53 ± 1.459	102.2 ± 3.594	44.91 ± 1.204	58.41 ± 1.755	17.01 ± 0.487
STZ-induced diabetic rats	204.3 ± 3.996^***^	205.6 ± 4.317^***^	34.43 ± 1.194^***^	161.6 ± 1.287^***^	33.94 ± 0.324^***^
STZ + High dose of the HELeKP	151.6 ± 1.646^###^	142.1 ± 2.249^###^	45.57 ± 0.763^###^	119.0 ± 0.473^###^	22.16 ± 0.688^###^
STZ + Low dose of the HELeKP	170.7 ± 2.142^###^	148.8 ± 2.282^###^	41.49 ± 0.1407^##^	127.7 ± 0.430^###^	25.01 ± 1.078^###^
STZ + Metformin	137.9 ± 2.544^###^	120.9 ± 1.684^###^	54.54 ± 1.153^###^	91.00 ± 0.429^###^	19.38 ± 0.076^###^

All values are expressed as mean ± SEM (Standard error of the mean)

***p < 0.001 as compared to the normal control rats

^##^*p* < 0.01, ^###^*p* < 0.001 as compared to STZ-induced diabetic rats

*NC* Normal control rats, *STZ* Streptozotocin, *HELeKP* Hydro-ethanolic leaf extract of *Koenigia polystachya*, *TC* Total cholesterol, *TG* Triglycerides, *HDL* High-density lipoprotein, *LDL* Low-density lipoprotein, *VLDL* Very low-density lipoprotein

#### Histopathological examination of pancreas and liver

**Fig. 2:**
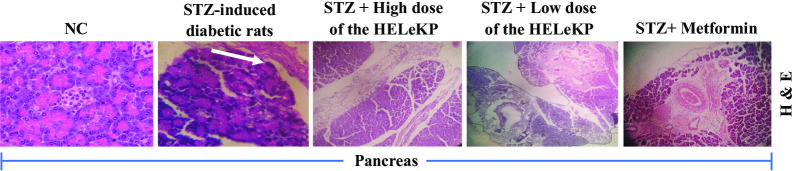
Representative histopathological analysis of different experimental pancreatic tissue of normal control (NC), Streptozotocin-induced diabetic rats (STZ), STZ + high dose of hydro-ethanolic leaf extract of *K. polystachya*, STZ + low dose of hydro-ethanolic leaf extract of *K. polystachya*, and STZ + Metformin obtained through Hematoxylin and Eosin (H and E) staining. Here, the white arrowhead indicates highly congested blood vessels and vacuolation of pancreatic cells (Magnification: 10X, except the NC and STZ groups having 20 ×)

The normal control rats didn’t show any abnormality in histological features of liver and pancreatic tissues. Whereas the STZ-treated rats showed severe clogging in the sinusoids, central vein (CV) and portal triad, along with the vacuolation and depletion of the pancreatic cells. Furthermore, the extract and standard-treated (metformin) group showed moderate congestion in the hepatocytic cells and a mild proliferation of bile ducts (Fig. 2), restoring the architecture of the liver and pancreas to normal.

## Discussion

The use of medicinal plants in India and China dates back centuries. One of these plants, *K. polystachya*, has been used by people since ancient times. Nagaland people also use this plant in their traditional cuisine. Since the dosage marks the medication as a poison, this may result in toxicity of the plant derivatives. Based on the OECD 425 toxicity guidelines (TG), one of this project's objectives was to evaluate the plant's toxicity via oral administration.

The clinical signs and symptoms can be used as indicators of toxic outcomes [12]. Following the limit dose of 2000 mg/kg b.w of rats, no animals died. The study detected no substantial alterations in body weight or food and water intake. All nutrients, comprising carbohydrates, proteins, and fats, are processed normally inside the body [13–15]. It has been shown that toxic substances are detrimental to the liver, brain, heart, and kidney of the body [16]. When the rats were sacrificed, no lesions or such observable changes were found on the histologic features of the liver, lung, heart, kidney, stomach, and spleen compared to the normal rats (Fig. 1).

The well-being of the body can also be measured using serum biomarkers, consisting of the liver serum markers (AST, ALT, ALP, and bilirubin levels), lipid profile markers (TC, TG) and kidney markers (urea and creatinine) [17–22]. Following the administration of the extract, there were no such substantial alterations in all the biochemical markers compared to the normal rats (Table 5), which also confirms the results with the histopathological reports. Moreover, any noxious stress or environmental impurity will alter hematological parameters [23]. There is no statistically significant change in all hematological counts, suggesting that cellular inflammation is not occurring.

The preliminary screening of the extract showed moderate antioxidant activity with the IC_50_ tabulated in Table 1. These free radicals are essential bioregulatory molecules that significantly develop chronic diseases like diabetes, arthritis, ulcerative colitis, Alzheimer’s, and more [24, 25]. A study by Vrchotova et al., reported that the extract from Himalayan Knotweed *Persicaria polystachya* (Meissner) H. Gross detected a high content of phenolic compounds, especially flavonols. Their study utilized the analytical techniques HPLC and CE (capillary electrophoresis) to confirm the presence of quercetin, catechin, quercetin-3-rhamnoside and quercetin-3-D-galactoside in the extract [9]. These compounds are considered for their anti-oxidant properties and also act as anti-diabetic agents [26, 27]. Aside from this, *K. polystachya* contains cardiac and saponin glycosides, which may contribute to its anti-diabetic properties. Studies suggest these could improve insulin sensitivity and glucose regulation [28]. Our *in-vitro* results also suggest that the HELeKP showed a concentration-dependent rise in the radical scavenging activity of leaf extract. Therefore, it might prevent the free radicals associated with diseases on regular consumption.

Streptozotocin causes damage to the pancreatic beta cells that secrete insulin. Furthermore, low insulin levels prevent cells from utilizing glucose, resulting in reactive oxygen species (ROS) [29]. The *K. polystachya* leaf extract significantly decreased blood sugar levels in diabetic rats, indicating its sufficient anti-diabetic properties. Because insulin largely controls glucose transport through the plasma membrane, it may restore the physical state of the plasma membrane [30].

It is common for DM patients to lose weight because their metabolic pathways are imbalanced. The current study found that STZ-rats administered with the HELeKP increased weight significantly, possibly due to reversed glycogenolysis and gluconeogenesis, and thus restored normal metabolic pathways [31, 32]. Moreover, diabetic rats had significantly higher lipid concentrations than control rats. Insulin deficiency can lead to metabolic and regulatory imbalances [33]. A histopathological study on diabetic rats showed that the extract supported these biochemical parameters by stimulating pancreatic beta-cell regeneration.

## Conclusions

Despite its antioxidant properties and traditional use for diabetes by the Nagaland people, *Koenigia polystachya* L. has not been scientifically proven to have an anti-diabetic effect. Hence, this study marks a significant contribution by addressing the scientific gap and contributing to further research. Given the acute toxicity studies performed per OECD 425, we can conclude that the extract is comparatively safe with a 2000 mg/kg *b.w.* oral dose. The preliminary results suggest, however, that repeated administrations would be examined for chronic toxicity studies. The experimental results demonstrate that the extract rejuvenates diabetic rats and has scavenging potential. To conclude, we need more precise approaches to exclude identifying its active biomolecules and the mechanism of action, which will surely be a new alternative to treat oxidative stress-related disorders.

## Methods

### Chemicals and drugs

The diabetogenic agent Streptozotocin (STZ) was purchased from Sisco-Research Laboratories (SRL) Pvt. Ltd., Mumbai, India. The standard Metformin hydrochloride was obtained as a gift sample from Angels Pharma India Pvt. Ltd. Hyderabad, India. 2,2-diphenyl-1-picrylhydrazine (DPPH), 2,2′-azino bis-(3-ethylbenzothiazoline-6-sulfonic acid (ABTS), Ascorbic acid and Trolox were purchased from Sigma Aldrich Chemicals Pvt. Ltd., Bangalore, India. All other chemicals used in this study were of analytical reagent grade obtained from standard commercial suppliers.

### Plant materials collection and extract preparation

The fresh leaves of the *Koenigia polystachya* L. plant were collected between September to October, 2021, specifically from Phesama village (Coordinates: 25.6260° N, 94.1079° E) in Kohima, Nagaland, Northeast India. After that, a botanist confirmed the plant specimen's authenticity at Kohima Science College, Jotsoma, Kohima, Nagaland, and retained it as a voucher specimen (GIPS/PHARMACOGNOSY/SPECIMEN NO.1) in the herbarium for the future. Subsequently, the plant sample was cleaned using regular water to eliminate soil and debris. It was then dried for 3–4 weeks in the shade at room temperature with optimum aeration. The size was decreased, ground well to powder form, and kept in a well-closed container in a dry place for extraction. The hydro-ethanolic (in the ratio of 1:3) extract was prepared by suspending the crushed leaves in a soxhlet extractor at 45 °C, followed by distillation to obtain a pure solid extract.

### Phytochemical analysis of the hydro-ethanolic leaf extract of K. polystachya

The procedure outlined by Khandelwal and Seck et al. [34, 35], was followed while screening the hydro-ethanolic leaf extract for several phytoconstituents.

### In-vitro anti-oxidant assay

#### DPPH radical scavenging antioxidant assay

The HELeKP assay followed the method by Vijayakumar et al. [36], with modifications. A 4 mL DPPH solution (100 µM) reaction mixture in 95% methanol was prepared. A leaf extract (HELeKP) stock solution was prepared at 1 mg/ml concentration and further diluted. The HELeKP has been studied at concentrations of 100, 200, 300, 400, and 500 µg/ml, which were incubated with the DPPH solution for 30 min at 37 °C. Absorbance at 517 nm was measured using a spectrophotometer, and ascorbic acid was used as the reference. The DPPH scavenging activity was expressed as IC50, and % inhibition was calculated using a formula. Readings were done in triplicate.

$$\% \;{\text{Inhibition}} = \frac{Control - Test}{{Control}} \times 100$$

#### ABTS radical scavenging antioxidant assay

The spectrophotometric estimation of the sample was conducted based on the method by Srinivasahan et al. [37], with a minor modification. A stock solution was prepared by combining equal amounts of 2.45 mM potassium persulfate and 7 mM ABTS aqueous solutions, then stored in the dark at room temperature for 12–16 h. The solution was diluted to achieve an absorbance of 0.706 ± 0.01 at 734 nm by mixing 2 ml of ABTS^+^ with 60 ml of methanol. Plant extract dilutions have been studied at concentrations of 100, 200, 300, 400, and 500 µg/ml, and absorbance was measured at 734 nm. Trolox served as the reference standard, and readings were taken in triplicate. The % inhibition of ABTS of the test sample was calculated using the formula mentioned for the DPPH.

#### H_2_O_2_ assay

The spectrophotometric evaluation of the H_2_O_2_ capacity of HELeKP was carried out based on the method by Gulcin et al. [38], with minor modifications. A 40 mM H_2_O_2_ solution in phosphate buffer (0.1 M, pH 7.4) was prepared. Serial dilutions of HELeKP have been studied at 100, 200, 300, 400, and 500 µg/ml concentrations. Following this, 2 ml of the H_2_O_2_ solution was added, and after a 10-min incubation, absorbance was measured at 230 nm. Ascorbic acid served as the reference, and triplicate readings were taken. The % inhibition of H_2_O_2_ of the test sample was calculated using the formula mentioned for the DPPH.

#### Superoxide radical scavenging activity

Based on Chetia et al. [39], the assay involved the conversion of nitroblue tetrazolium (NBT) to NBT diformazan by the superoxide radical. HELeKP at various concentrations of 100, 200, 300, 400, and 500 µg/ml was mixed with NBT (156 M), NADH (468 M), and phenazine methosulphate (100 ml). The reaction mixture was incubated at 25 °C for 5 min, and absorbance was measured at 560 nm using a spectrophotometer. Ascorbic acid served as the [reference, and triplicate readings were taken. The % inhibition of superoxide of the test sample was calculated using the formula mentioned for the DPPH.

#### Nitric oxide assay

The Chetia et al*.* [39], technique was adopted to generate nitric oxide (NO) from sodium nitroprusside via the Griess reaction. HELeKP at different concentrations of 100, 200, 300, 400, and 500 µg/ml was mixed with sodium nitroprusside (5 mM) in phosphate-buffered saline and incubated at 25 °C for 150 min. Griess reagent was used to measure absorbance at 546 nm. Ascorbic acid served as the reference and triplicate readings were taken. The % inhibition of NO of the test sample was calculated using the formula mentioned for the DPPH.

### In-vitro anti-diabetic assay

#### α-amylase inhibitory assay

The α-amylase assay was adapted from Wickramaratne et al. [40], with slight modifications. HELeKP at various concentrations of 20, 40, 60, 80, and 100 μg/ml was mixed with 10% DMSO and buffer solution (pH 6.9). The mixture was incubated with the α-amylase solution for 10 min at 30 °C, followed by adding starch solution. The reaction was stopped with 3,5-Dinitrosalicylic acid (DNSA) reagent, and absorbance was measured at 540 nm. Acarbose was used as a reference, and triplicate readings were taken. The % inhibition was calculated using the DPPH formula.

#### α-glucosidase enzyme inhibition assay

The α-glucosidase assay was adapted from Abbas et al. [41], with modifications. HELeKP at 20, 40, 60, 80, and 100 μg/ml concentrations was incubated with α-glucosidase enzyme solution, phosphate buffer (pH 6.8), and p-nitrophenyl glucopyranoside (PNPG) for specific durations. The reaction was stopped, and absorbance was measured at 405 nm. Ascorbic acid served as the reference, and triplicate measurements were taken.

#### Animal experiments

The study employed Wistar male albino rats weighing 150–200 g. The animals were caged under normal laboratory conditions, a 12-h light and dark cycle, a temperature of 27 ± 2 °C, and fed with a standard rodent diet and water ad libitum. After obtaining the required consent from the Institutional Animal Ethics Committee (IAEC), the study was carried out by the CCSEA standards (Approval No. GIPS/IEAC/M.PH/PRO/13/2021).

#### Acute oral toxicity assay

The rats were arbitrarily selected, marked for identification, and housed in their cages for acclimatization. Before dosage, the rats were kept fasted overnight but had access to water. The animals were distributed into four groups (n = 5), and the limit test was performed at a single dose of 2000 mg/kg *p.o* for the HELeKP as per the OECD guidelines 425, as tabulated in Table 9 [17]. The animals were carefully monitored for 30 min, followed by the next 4 h. The food pellet was supplied after 1–2 h of the extract administration. After the treated rats survived, four new animals were given the same treatment dose (2000 mg/kg b.w. *p.o*). All the animals were carefully monitored for the first 6 h and subsequently at regular gaps for 14 days. The rats' weight was closely monitored from the start of the study. Following the end of the study, blood/serum samples collected from the overnight fasted rats after anesthetization (Ketamine and xylazine) were taken for different hematological and biochemical estimations using standard available marketed kits. Later, the vital organs were collected for morphological and histopathological changes.

**Table 9:** Grouping of animals (male and female) for acute oral toxicity study

Grouping and Treatment	Gender (n = 5)
Group I: Vehicle control (0.3% CMC)	Male
Group II: Vehicle control (0.3% CMC)	Female
Group III: 2000 mg/kg *b.w.* of plant extract	Male
Group IV: 2000 mg/kg *b.w.* of plant extract	Female

### Experimental protocol (Streptozotocin-induced diabetes model)

The experimental animals for the anti-diabetic activity were divided into five groups (n = 8), as given in Table 10 [42, 43]. All the animals were documented for any alteration in body weight before (day 0) and throughout the treatment period (on the 7th, 14th and 21st). The blood/serum for different biochemical estimations samples was collected after anesthetization (Ketamine and xylazine). Later, the tissue samples were collected from the euthanized animals after 21 days of the study for histopathological observation.

**Table 10:** Experimental design and treatment protocol for the anti-diabetic study [42, 43]

Grouping	Treatment
I	Normal control rats (NC); received carboxymethyl cellulose (CMC) of 0.3% w/v *p.o* for 21 days
II	Diabetic control rats (STZ); received a single injection of streptozotocin (STZ; 55 mg/kg *b.w. i.p*.) prepared in 0.05 M ice-cold citrate buffer solution at pH 4.5
III	Treatment (STZ + High dose of the HELeKP); following the confirmation of the increased blood glucose levels, this group received the hydro-ethanolic extract of the plant at a dose of 200 mg/kg *b.w. p.o.* once daily for 21 days
IV	Treatment (STZ + Low dose of the HELeKP); following the confirmation of the increased blood glucose levels, this group received the hydro-ethanolic extract of the plant at a dose of 66.6 mg/kg *b.w. p.o.* once daily for 21 days
V	Standard (STZ + Metformin); following the confirmation of the increased blood glucose levels, this group received the standard metformin at a dose of 5 mg/kg *b.w. p.o.* once daily for 21 days

### Biochemical analysis

The measurement of the serum fasting glucose, liver function markers (AST, ALT, ALP, and bilirubin), renal function markers (Urea and creatinine) and lipid profile levels [triglycerides (TG), total cholesterol (TC), high-density lipoprotein (HDL), low-density lipoprotein (LDL) and very low-density lipoprotein (VLDL)] were measured as per the instruction of the standard commercial kits using Microlab 300 semi-automated analyzer. Furthermore, the hematological parameters for all the experimental groups were carried out using the standard kit incorporated using the Erba Transasia H360 hematology analyzer.

### Histopathological analysis

After fixing the tissues, isopropyl alcohol (60–100%) was used to dehydrate the tissues and set them in paraffin wax. A light microscope was used to capture images of the sectioned slides after they were stained with hematoxylin and eosin (H and E).

### Statistical analysis

The result obtained from several investigational groups was calculated as mean ± Standard error of the mean (SEM) using GraphPad Prism version 6.0 statistical software. The outcomes were expressed by adopting a one-way ANOVA (Analysis of Variance), followed by Bonferroni’s multiple comparison tests as post-hoc, wherever applicable. The value of *p* < 0.05 was measured as statistically significant.

## Data Availability

Not applicable.

## Abbreviations

ABTS: 2,2′-Azino bis-(3-ethylbenzothiazoline-6-sulfonic acid)
AST: Aspartate Transaminase
ALT: Alanine Transaminase
ALP: Alkaline Phosphatase
CCSEA: Committee for the Control and Supervision of Experiments on Animals
DPPH: 2,2-Diphenyl-1-picrylhydrazine
HELeKP: Hydro-ethanolic leaf extract of *Koenigia polystachya*
H_2_O_2_: Hydrogen peroxide
IC50: Half-maximal inhibitory concentration
NO: Nitric oxide
OECD: Organization for Economic Cooperation and Development
STZ: Streptozotocin
